# Whole Genome Wide Expression Profiles on Germination of *Verticillium dahliae* Microsclerotia

**DOI:** 10.1371/journal.pone.0100046

**Published:** 2014-06-13

**Authors:** Dongfang Hu, Chunsheng Wang, Fei Tao, Qian Cui, Xiangming Xu, Wenjing Shang, Xiaoping Hu

**Affiliations:** 1 State Key Laboratory of Crop Stress Biology for Arid Areas, College of Plant Protection, Northwest A & F University, Yangling, China; 2 East Malling Research, East Malling, Kent, United Kingdom; Yonsei University, Republic Of Korea

## Abstract

*Verticillium dahliae* is a fungal pathogen causing *Verticillium* wilt on a range of economically important crops. Microsclerotia are its main survival and dormancy structures and serve as the primary inoculum on many hosts. Studies were conducted to determine the effect of temperature (5 to 50°C), pH (2 to 12) and nutrient regimes on microsclerotia germination. The optimal condition for microsclerotium germination was 20°C with pH 8.0 whereas nutrient regimes had no significant effect on its germination. The whole genome wide expression profiles during microsclerotium germination were characterized using the Illumina sequencing technology. Approximately 7.4 million of 21-nt cDNA tags were sequenced in the cDNA libraries derived from germinated and non-germinated microsclerotia. About 3.9% and 2.3% of the unique tags were up-regulated and down-regulated at least five-fold, respectively, in the germinated microsclerotia compared with the non-germinated microsclerotia. A total of 1654 genes showing differential expression were identified. Genes that are likely to have played important roles in microsclerotium germination include those encoding G-protein coupled receptor, lipase/esterase, cyclopentanone 1,2-monooxygenase, H(+)/hexose cotransporter 1, fungal Zn(2)-Cys(6) binuclear cluster domain, thymus-specific serine protease, glucan 1,3-beta-glucosidase, and alcohol dehydrogenase. These genes were mainly up-regulated or down-regulated only in germinated microsclerotia, compared with non-germinated microsclerotia. The differential expression of genes was confirmed by qRT-PCR analysis of 20 randomly selected genes from the 40 most differentially expressed genes.

## Introduction

*Verticillium dahliae* Kleb. is a soilborne fungal pathogen causing vascular wilt disease on more than 160 plant species, including many agriculturally important crops [1]. Microsclerotia, the primary inoculum of *V. dahliae*, can survive for more than 10 years in soil [1]. Microsclerotia are a specialized structure and are resistant to harsh conditions, ensuring the survival of *V. dahliae*. They are composed of compact masses of thick-walled, pigmented cells, which originate from swollen, septate hyphae by a process of budding [2]. Reducing the density of viable microsclerotia in soil is one of key methods for effective management of *Verticillium* wilt diseases. Usually, such disease control is managed through soil fumigation with methyl bromide; however this chemical was recently banned from commercial use because of its harmful effects on human health and stratospheric ozone, and other soil microorganisms.

Microsclerotium development of *V. dahliae* has been extensively studied [3]–[5]. A hydrophobin gene (*VDH1*) was cloned and shown to be necessary for microsclerotium production. This gene mediates the development of microsclerotia from conidiophores and other hyphal structures, and its activity appears to be regulated in response to carbon availability [4]. The gene expression in the microsclerotia of *V. dahliae* was analyzed through RNA-seq to shed molecular processes on microsclerotium biogenesis and melanin synthesis [6].

Germination of microsclerotia is the first step in microsclerotia returning to vegetative growth and initiating wilt diseases, and is a key process that can be targeted to reduce the survival of *V. dahliae* inoculum in soil [7]. A single microsclerotium may germinate up to 13 times, with the number of repeated germination events positively correlated with the microsclerotium size [8]. Germination of microsclerotia is independent of external nutrient supply but depends on the status of microsclerotia and availability of free water [8], and is also stimulated by root exudates of both host and non-hosts. Although much effort has been directed to identify genes involved in specific stages of fungal development [4], [9], [10], molecular mechanism(s) responsible for the germination of *V. dahliae* microsclerotia remains to be elucidated. Understanding such mechanism(s) may be useful for developing novel strategies to control wilt diseases.

The paper reports results from experiments on conditions necessary for microsclerotium germination and on development of cDNA libraries during the microsclerotium germination, which may be used for future study of molecular mechanisms controlling the germination process. Specific objectives were to (1) determine the effect of temperature, pH values and nutrient regimes on microsclerotium germination, (2) build a comprehensive database of genes that are differentially regulated during microsclerotium germination, and (3) identify genes for future functional analysis.

## Materials and Methods

### Fungal strain maintenance

A single strain of *V. dahliae* (XJ2008), a defoliating pathotype, was used; this strain was isolated from an infected cotton plant in Xinjiang province, China in 2008 (Fig. S1). The basal modified medium (BMM) was used for microsclerotium production; BMM was composed of glucose, 5.0 g/L; NaNO_3_, 0.2 g/L; KCl, 0.5 g/L; MgSO_4_·7H_2_O, 0.5 g/L; KH_2_PO_4_, 1.5 g/L; vitamin B1, 3 µM; vitamin H, 0.1 µM; and agar, 15.0 g/L. Deionized water was used and the pH was adjusted to 11.5 using sodium hydroxide [11]. The strain was inoculated onto a sterile cellophane disc (HC273, Nanjing Oddfoni Biological Technology Co., Ltd., China) that covers BMM medium in a 9 cm Petri dish and incubated at 20°C in dark for 30 days.

### Harvesting microsclerotia

A sterile blade was used to scrape the 30-days-old culture to harvest microsclerotia. The fungal materials were collected using a 500 ml beaker, comminuted with a blender (HR2839, Philips) in 200 ml of tap water for four 10-second pulses, then poured through a nested set of Tyler standard sieves of mesh sizes 150 (upper layer) and 400 (lower layer) (corresponding to the opening of 106 µm and 38 µm, respectively). The content on the sieves were rinsed under tap water for 2 min; the remaining content on the bottom layer were washed back into the beaker and comminuted again for four 10-second pulses. A sample of microsclerotia was assessed on a slide under a stereo dissecting microscope between each rinse to determine whether further blending was needed. This process was repeated until the microsclerotia on the bottom layer sieve were singular and free from mats of hyphal fragments. In the final wash, microsclerotia were washed off the sieves with a minimal volume of water (2–10 ml), and transferred into several 1.5 ml eppendorf tubes. The tubes were centrifuged at 5000 rpm for 5 min, and the supernatant was discarded. The pellet was kept at 47°C for 5 min in water bath to kill mycelia and conidia but without harming microsclerotia [12]. Finally, the tubes were air-dried inside an incubator (47°C) for 12 h and then stored at −80°C until needed.

### Conditions for microsclerotium germination

The effect of temperature (5–50°C) and pH (2–12) and nutrient regime on microsclerotium germination was investigated. Germination at ten temperatures (5, 10, 15, 20, 25, 30, 35, 40, 45, 50°C) was studied. One hundred microsclerotia were randomly picked with a botanical needle under a stereo dissecting microscope and placed onto a concave slide with deionized water, which was covered with a cover slip and kept for 16 h in dark in an incubator set to one of the ten temperatures. Similarly, 100 microsclerotia were placed into deionized water (ca. 0.5 ml) adjusted to one of 11 pH values (2, 3, 4, 5, 6, 7, 8, 9, 10, 11 or 12) and incubated in dark for 16 h at 20°C. The pH value was adjusted with sodium hydroxide and hydrochloric acid. For nutritional effects on microsclerotium germination, 100 microsclerotia were placed into ca. 0.5 ml 2% (w/v) of each of the following solutions: glucose, sucrose, maltose, mannitol, rhamnose, fructose, lactose and arabinose; solutions with microsclerotia were incubated in dark for 16 h at 20°C. Number of germinated microsclerotia was counted under a microscope after 16 h of incubation [13]. A microsclerotium was considered to have germinated when the germ tube is longer than the microsclerotium diameter. All experiments were repeated three times. The Student-Newman-Keuls test was used to compare treatment effects on microsclerotium germination after ANOVA analysis where % germination was arcsine-transformed.

### RNA extraction and digital expression library preparation

**Figure 1:**
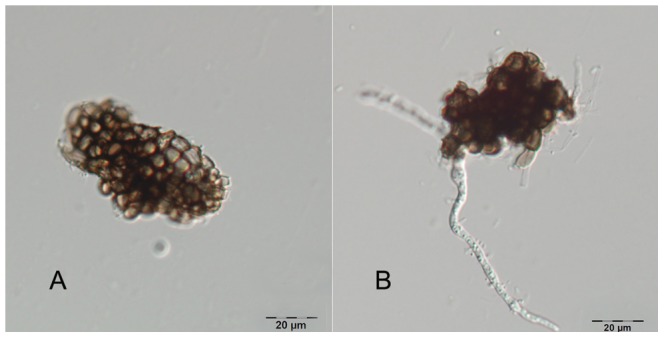
A non-germinated (A) and germinated (B) microsclerotia of *Verticillium dahliae*.

Microsclerotia were collected and divided into six equal parts (approximately 3 g each). Microsclerotia in three of the six parts were used as control and kept at −80°C; microsclerotia in the other three parts were incubated for 16 h under the optimum condition for germination, namely, deionized water with pH adjusted to 8.0 at 20°C in dark. Microsclerotia from both the control (Fig. 1 A) and the germination treatment (Fig. 1 B) were harvested after centrifugation at 5000 rpm for 5 min. Total RNA was extracted from germinated microsclerotia for library construction using standard protocols (Trizol) and then treated with DNase to remove potential genomic DNA contamination according to the manufacturer's protocols. Trizol, DNase and high pure RNA Isolation Kit were obtained from Roche Applied Science (Mannheim, Germany). In parallel, non-germinated microsclerotia from the control were processed identically. RNA integrity and concentration were evaluated (2100 Bioanalyzer, Agilent Technologies). RNA from the three non-germinated *V. dahliae* microsclerotia samples and the three germinated *V. dahliae* microsclerotia samples was pooled separately (Table S1). Sequence tag preparation was carried out using the Digital Gene Expression Tag Profiling Kit (Illumina Inc, USA) according to the manufacturer's protocol (version 2.1B). Six micrograms of total RNA was extracted and mRNA was purified using biotin-Oligo (dT) magnetic bead adsorption. First- and second-strand cDNA synthesis was performed after the RNA was bound to the beads. Double stranded cDNA was digested with the *Nla*III endonuclease to produce a bead-bound cDNA fragment containing sequence from the 3′-most CATG to the poly (A)-tail, and these 3′ cDNA fragments were purified using magnetic bead precipitation. The Illumina adapter 1 (GEX adapter 1) was added to the new 5′-end. The junction of Illumina adapter 1 and CATG site was recognized by *Mme*I, a Type I endonuclease (with different recognition and digestion sites). *Mme*I cuts 17 bp downstream of the CATG site, producing 17 bp cDNA sequence tags with the adapter 1. After removing 3′ fragments with magnetic bead precipitation, the Illumina adapter 2 (GEX adapter 2) was ligated to 3′-end of the cDNA tag. These cDNA fragments represented the tag library.

### Illumina sequencing

Illumina Genome Analyzer platform (GA II) was used to perform a Serial Analysis of Gene Expression (SAGE)-derived Digital Gene Expression (DGE) analysis for the transcriptome of germinated and non-germinated *V. dahliae* microsclerotia (BGI, China). To enrich the desired DNA fragments, samples were amplified for 15 PCR cycles using Phusion polymerase (Finnzymes, Espoo, Finland) with primers complementary to the adapter sequences. The resulting 85 base strips were purified by 6% TBE PAGE gel electrophoresis and then digested. The single chain molecules were fixed onto the Illumina Sequencing Chip (flow cell). Each molecule grew into a single-molecule cluster sequencing template through *in situ* amplification. Four color-labeled nucleotides were added, and sequencing was performed (HuaDa Gene, http://www.genomics.org.cn/) via the sequencing by synthesis method. Image analysis and base calling were performed using the Illumina Pipeline; cDNA sequence tags were revealed after purity filtering. The tags passing initial quality tests were sorted and counted. Each channel generated millions of raw reads with sequencing length of 35 bp (target tags plus 3′ adaptor). Each molecule in the library represented a single tag derived from a single transcript.

### Sequence annotation

Sequenced tags were first filtered to remove those low-quality or adaptor-only tags. The remaining clean tags were subsequently analyzed to estimate their copy number and hence their percentage in the total clean tags. The BlastN program was used to align the Tag sequences against the NCBI database (http://blast.ncbi.nlm.nih.gov/Blast.cgi) and *V. dahliae* genome database (http://www.broadinstitute.org/annotation/genome/verticillium_dahliae) [14]. All clean tags were annotated based on *V. dahliae* reference genes. For conservative and reliable annotation, only sequences with perfect homology or only 1 nt mismatch were considered further. The number of annotated clean tags for each gene was estimated and normalized to TPM (number of transcripts per million clean tags) [15]–[17]. Each sequence was assigned to a functional category based on the literature and the Kyoto Encyclopedia of Genes and Genomes (KEGG) database.

### Identification of differentially expressed genes (DEGs)

After adaptor and quality trimming, the clear reads were used for downstream bioinformatics analysis. Sequencing saturation was performed to check whether the number of detected genes keep increasing when sequencing amount (total tag number) increases. Alignment was performed using Bowite (http://bowtie-bio.sourceforge.net/index.shtml) [18]. Gene expression was calculated using TPM method with IDEG6 [19], [20]. The TPM was normalized and directly used for subsequent analysis. DEGseq packages were used to identify differentially expressed genes between VDMG-b and VDM libraries. *P* values in the experiment without biological repeats were calculated using Fisher's exact test by DEGseq software [21], [22]. Histogram for the number of reads for genes of VDM (Fig. S2A) and VDMG-b (Fig. S2B), Boxplot (Fig. S2C) and scatter plot (Fig. S2D) were generated based on log2 (read counts for each gene). The criteria of ‘FET *P*<0.001 [23] and the absolute value of log_2_^Ratio^ ≥1′ (Ratio: Ratio of the expressed tag frequency between VDMG-b and VDM libraries) were used to judge the statistical significance of differential expression for one particular gene between samples.

### Quantification of differentially expressed genes

RNA of germinated and non-germinated microsclerotia from three biological replicates was extracted as described above for Illumina sequencing. The genomic DNA was eliminated by treatment at 42°C with gDNA Eraser for 2 min. The reverse transcriptase reaction was then carried out at 37°C for 15 min and 85°C for 5 sec using Prime Script RT reagent Kit with gDNA Eraser (TaKaRa, Japan). To confirm the transcriptomic results, the expression levels of 20 genes (Table 1), which were most differentially expressed between germinated and non-germinated microsclerotia, were randomly selected from the 40 most differentially expressed genes and quantified using the quantitative real-time reverse transpcription PCR (qRT-PCR). A stringent set of criteria was used for primer design, including predicted melting temperatures of 80–90°C, limited self-complementarity, primer length between 18 and 24 nt, and PCR amplicon length of 50 to 200 bp. qRT-PCR reactions were done in a final volume of 20 µL containing 1 µL cDNA of *V. dahliae* microsclerotia, 10 µL of 2× SYBR Green I (Bio-Rad, USA) and 400 nM of forward and reverse primers (Table 2). The thermal cycling conditions were one cycle of 95°C for 3 min, 40 cycles of 95°C for 15 s for denaturation, temperature as shown in Table 2 for 15 s for annealing, and 72°C for 30 s for extension. Reactions were run for the three biological replicates of each treatment; the presence of dimer can be assessed from dissociation curves. qRT-PCR efficiency was determined by a series of 2-fold dilutions of cDNA. The calculated efficiency of all primers was 0.9–1.0. Beta-tubulin gene (accession no. DQ522531) of *V. dahliae* was used for calibration in all experiments. The expression ratio of each gene was calculated from cycle threshold (C_T_) values using 2^−**ΔΔ**C_T_^ method (24). Briefly, the expression levels of each genes and beta-tubulin gene (an endogenou reference control gene) were evaluated both in germinated *V. dahliae* microsclerotia (VDM) and non-germinated *V. dahliae* microsclerotia (VDMG-b). Then, the ΔΔCT values were calculated by:

**Table 1:** Forty genes that showed most differential expression between the germinated (VDMG-b) and non-germinated (VDM) microsclerotia of *Verticillium dahliae*.

Gene	Annotation	Relative abundance^a^	*P*-Value^b^	Chromosome	Gene confirmed by qRT-PCR	Reference gene length (nt)
Up-regulated genes						
Metabolism						
VDAG_03943	cyclopentanone 1,2-monooxygenase [*Physcomitrella patens* subsp. *patens*]	14.9717	9.63E-84	Chr3	Yes	1929
VDAG_05094	FAD binding domain-containing protein [*Selaginella moellendorffii*]	11.8719	2.43E-9	Chr4		1592
VDAG_07040	lipase/esterase	11.1241	1.20E-05	Chr5		1587
VDAG_03555	thymus-specific serine protease[*Phytophthora infestans* T30-4]	11.1241	1.20E-05	Chr6	Yes	1727
VDAG_03505	glutathione-dependent formaldehyde-activating GFA [*Micromonas pusilla* CCMP1545]	10.8611	1.35E-04	Chr6		522
VDAG_02814	glucan 1,3-beta-glucosidase	10.3310	3.58E-12	Chr3	Yes	2464
VDAG_02566	short chain alcohol dehydrogenase [*Ricinus communis*]	10.2644	1.19E-11	Chr3	Yes	1089
VDAG_02101	fatty acid hydroxylase, cytochrome P450 [*Thalassiosira pseudonana* CCMP1335]	10.1960	3.99E-11	Chr7	Yes	1734
Transcription						
VDAG_05553	Fungal Zn(2)-Cys(6) binuclear cluster domain	11.5108	1.58E-26	Chr2	Yes	1552
VDAG_07325	Zn_clus Fungal specific transcription factor domain	10.9680	1.91E-18	Chr5	Yes	2934
VDAG_00261	IDI-3 protein	11.4823	5.85E-26	Chr2		618
Transport						
VDAG_07083	H(+)/hexose cotransporter [*Solanum lycopersicum*]	13.2136	2.31E-85	Chr5	Yes	2065
Signal transduction						
VDAG_04611	G-protein coupled receptor	10.3934	1.94E-12	Chr3	Yes	1618
Unknown function						
VDAG_09753	hypothetical protein	11.9780	1.50E-36	Chr3	Yes	842
VDAG_06930	conserved hypothetical protein	11.9046	7.19E-35	Chr5		1366
VDAG_05278	hypothetical protein	11.1605	4.25E-21	Chr4		770
VDAG_09977	conserved hypothetical protein	10.5934	2/17E-14	Chr4		2133
VDAG_09105	hypothetical protein	10.5934	2.17E-14	Chr4		1150
VDAG_05341	conserved hypothetical protein	10.5661	4.06E-14	Chr2		1831
VDAG_10462	conserved hypothetical protein	10.4533	5.23E-13	Unpositioned scaffolds		969
Down regulated gene						
Metabolism						
VDAG_07578	FAD binding domain-containing protein	−10.9366	1.21E-23	Chr5	Yes	1548
VDAG_08069	mannose-6-phosphate isomerase	−10.3061	1.55E-15	Chr6	Yes	1273
VDAG_10195	vacuolar protein sorting-associated protein	−10.0014	1.03E-12	Unpositioned scaffolds	Yes	9752
VDAG_00511	glucan 1,3-beta-glucosidase	−10.0014	1.03E-12	Chr2		1610
VDAG_02457	sulfate adenylyltransferase	−9.8688	1.18E-11	Chr3	Yes	1943
VDAG_10268	pectinesterase	−9.5584	1.53E-09	Unpositioned scaffolds	Yes	7590
VDAG_08889	NADH-ubiquinone oxidoreductase 78 kDa subunit	−9.4999	3.45E-09	Chr5		2549
VDAG_10441	cortical actin cytoskeleton protein asp1	−9.4999	3.45E-09	Unpositioned scaffolds		5249
VDAG_00349	SAP domain-containing protein	−9.2360	8.88E-08	Chr2	Yes	1989
VDAG_03333	chitin deacetylase	−9.1624	2.00E-07	Chr6	Yes	1380
Transport						
VDAG_02707	pleiotropic ABC efflux transporter of multiple drugs	−12.0227	2.20E-49	Chr3	Yes	4827
VDAG_08272	mitochondrial phosphate carrier protein	−9.2360	8.88E-08	Chr6		991
Transcription						
VDAG_07479	DNA damage-responsive transcriptional repressor RPH1	−9.0028	1.01E-06	Chr5	Yes	5594
VDAG_03711	WSC domain-containing protein	−10.1624	4.00–14	Chr3	Yes	1980
Unknown function						
VDAG_03584	predicted protein	−9.8688	1.18E-11	Chr3		1062
VDAG_07143	conserved hypothetical protein	−9.7715	5.96E-11	Chr5		3580
VDAG_09013	conserved hypothetical protein	−9.7211	1.34E-10	Chr5		952
VDAG_07396	conserved hypothetical protein	−9.3061	3.94E-08	Chr5		1232
VDAG_00594	conserved hypothetical protein	−9.0848	4.50E-07	Chr2		1064
VDAG_10196	conserved hypothetical protein	−9.0028	1.01E-06	Unpositioned scaffolds		1511

a The relative abundance was expressed as log_2_^Ratio(VDMG-b/VDM)^, where VDMG-b/VDM was the ratio of the transcripts per million clean tags between the VDMG-b and VDM libraries.

b Probability value between VDMG-b and VDM libraries was calculated using Fisher's exact test.

**Table 2:** Primers for qRT-PCR quantification of 20 randomly selected genes from the 40 most differentially expressed genes that were up-regulated or down-regulated in the germinated *Verticillium dahliae* microsclerotia (VDMG-b) compared to the non-germinated *Verticillium dahliae* microsclerotia (VDM).

Accession number	Molecular function	Primer (5′-3′)	Amplification Length (bp)	Annealing temperature (°C)	Efficiency (%)
VDAG_07083	H(+)/hexose cotransporter	F:CGGTTCTATCTGCTACGC R:TACCCTTCCCGGTTGAG	152	63	99.9
VDAG_05553	Fungal Zn(2)-Cys(6) binuclear cluster domain	F:CGAGGAATACGAGACCG R:ATGGAACGCAGACAACG	126	62	102.8
VDAG_03943	Cyclopentanone 1,2-monooxygenase	F:AGTCGCCGAGCAAGAGC R:TCCAGTCGTAGTGAAACCC	89	65	97.0
VDAG_07578	FAD binding domain-containing protein	F:CGTGCTTTGAGCCGTTCT R:AGTCGGCCACCGTCTTG	69	67	104.9
VDAG_03333	Chitin deacetylase	F:ATGGTTGGGAAGAATGGA R:TGGGATGGCTGAATGAGT	112	61	97.7
VDAG_03711	WSC domain-containing protein	F:CTCAGTGTTCTTCCTGGTGA R:CCGTTCTTGGTTCCGTAG	59	63	100.6
VDAG_02566	short chain alcohol dehydrogenase	F:CGGGCTGGTTCCCTTTGT R:TGTTGGAGCCGCTGATGAA	146	57	101.2
VDAG_02814	glucan 1,3-beta-glucosidase	F:GATGCGTTCCTGGTGTCTG R:CCTTGATGACGGGCAGAT	147	55	97.1
VDAG_02707	pleiotropic ABC efflux transporter of multiple drugs	F:TCGCCTCGTTCTGGACC R:CCTTGAAGACTATTGCTGGATGT	154	57	99.3
VDAG_08069	mannose-6-phosphate isomerase	F:GCGACTACCCAGACTTTCCT R:GTTCCTTGTTCGGGTGTATCT	191	56	104.6
VDAG_02457	sulfate adenylyltransferase	F:AAGCCTGGTGACATTGAC R:TGGTTCTTGCGGATGATG	158	60	97.6
VDAG_07479	DNA damage-responsive transcriptional repressor RPH1	F:CGACGAACAACAATCCATT R:GACCAGAAGAGACAGAATACT	87	62	103.7
VDAG_04611	G-protein coupled receptor	F:ACACTGCGAGGAAGGTTA R:AAGGTGACAATGATGAATAGACA	112	60	104.3
VDAG_07325	Zn_clus fungal specific transcription factor domain	F:CAGAGTATCCATCCAGGTC R:CGTGTAGTATCGGGTAGAG	174	63	102.3
VDAG_03555	thymus-specific serine protease	F:GTAGAAGATGTCACTCAGAAC R:ATCGTCAAACCAGTATCGTA	189	63	98.3
VDAG_02101	fatty acid hydroxylase, cytochrome P450	F:GACTACAACATCCTAACGCTACC R:CAGAAGAACGCCTCGCATAG	93	60	97.3
VDAG_09753	hypothetical protein	F:TTGACTCCGACGACGATGCT R:CGACTGCTACGATGCCTCCTA	117	62	96.5
VDAG_10195	vacuolar protein sorting-associated protein	F:AGTTCCTCATTAGTTCGTTCAC R:GGCTATCCACCGTTACCA	168	65	103.1
VDAG_00349	SAP domain-containing protein	F:CCTCTCAAGCCAACTCCATA R:CGTATCGTATCTCCCACCTT	270	63	96.0
VDAG_10268	pectinesterase	F:CATCAACCTCGTCAACAC R:CAACCGTAGAAGCCAATC	99	60	105.9
VDAG_10074	Tubulin beta chain	F:TTTCCAGATCACCCACTCC R:ACGACCGAGAAGGTAGCC	114	60	101.1

ΔΔCT  =  ΔCT VDMG-b.gene - ΔCT VDM.gene

Where, ΔCT VDMG-b.gene  =  CT VDMG-b.gene - CT beta-tubulin

ΔCT VDM.gene  =  CT VDM.gene - CT beta-tubulin

If the value of 2^−**ΔΔ**CT^ was greater than 1.0, it meant the gene expression level in VDMG-b was higher than that in VDM (the gene expression was up-regulated). If the value of 2^−**ΔΔ**CT^ was less than 1.0, it meant the gene expression level in VDMG-b was lower than that in VDM (the gene expression was down-regulated). The relative expression values of those 20 genes were then used to validate for the RNA-seq data through correlation analysis.

## Results

### Conditions for microsclerotium germination

**Figure 2:**
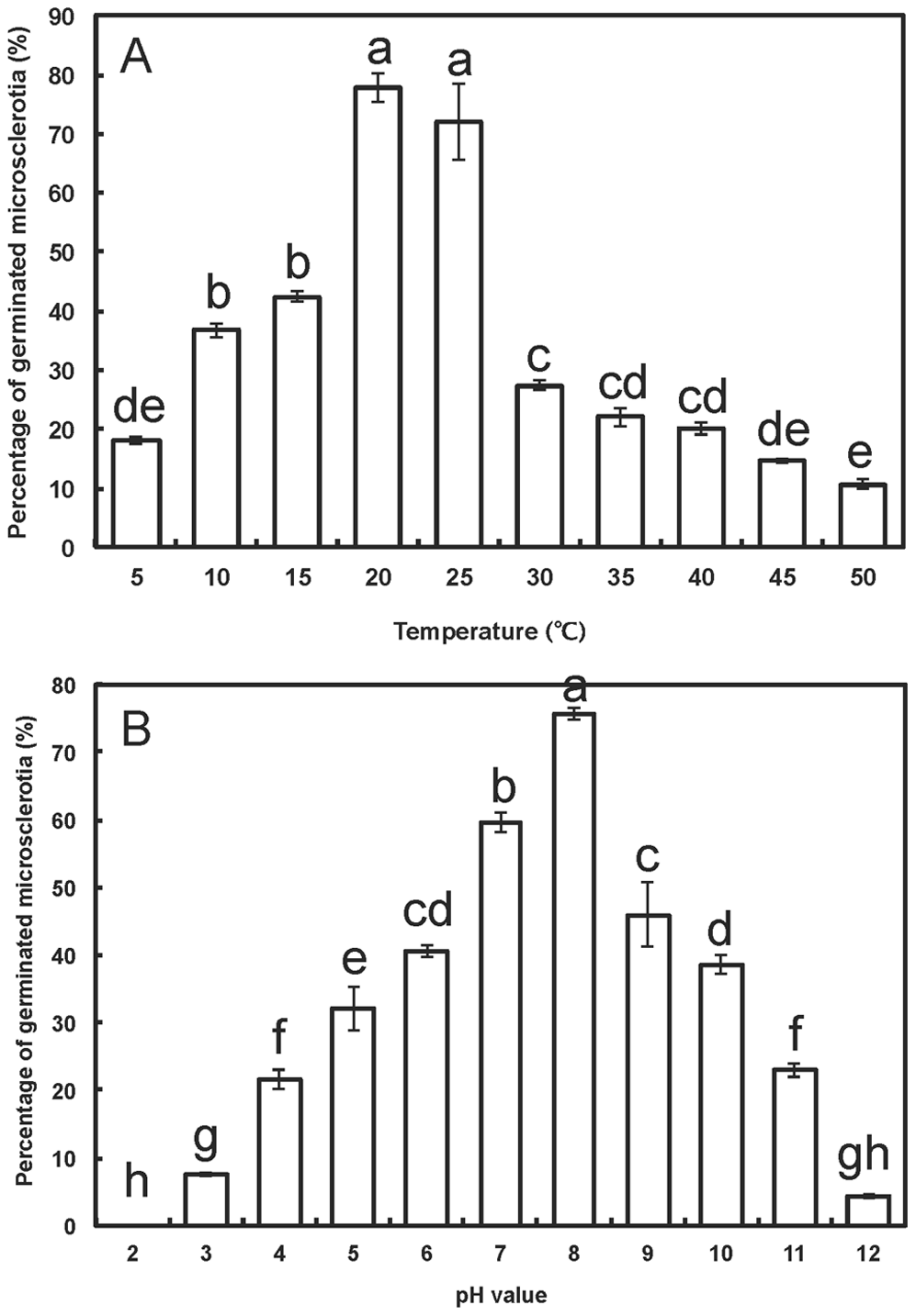
Percentage of germinated *Verticillium dahliae* microsclerotia after 16 h incubation in darkness at different temperatures (A) and pH (B). Data are shown as the mean of three replicated expriments. Bars represent standard error (SE) of means. Bars marked with different letters (lowercase) are significantly different (*P*<0.05).

Percentage of germinated microsclerotia varied greatly with temperature and pH (Fig. 2). Microsclerotia showed the highest germination of 78% at 20°C, and the lowest germination of 12% at 50°C (Fig. 2A), as well as the highest germination of 76% at pH 8.0 (Fig. 2B). Germination of microsclerotia was not significantly affected by the nutrients studied; the level of germination in different nutrients was comparable to that observed in sterile deionized water.

### Total gene expression profile

**Figure 3:**
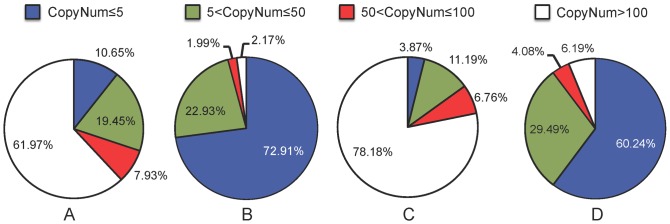
Frequency distribution of total tag and distinct tag counts over different tag abundance categories from the two libraries of non-germinated (VDM) and germinated (VDMG-b) *Verticillium dahliae* microsclerotia. A, distribution of total clean tags in the non-germinated microsclerotia (VDM); B, distribution of VDM distinct clean tags; C, distribution of total clean tags in the germinated microsclerotia (VDMG-b); D, distribution of VDMG-b distinct clean tags.

RNA-seq raw tags of *V. dahliae* microsclerotia were deposited in NCBI (http://www.ncbi.nlm.nih.gov/) and can be accessed in the SRA (VDMG-b with accession no. SRR974961, and VDM with accession no. SRR975062). In total, there were 3,671,123 and 3,733,211 tags sequenced in the VDMG-b and VDM libraries, respectively (Table 3); there were corresponding 3,495,080 and 3,316,376 clean tags, containing 81,034 and 186,721 distinct tags respectivley. The frequency distribution of distinct clean tags as well as total clean tags varied considerably between the two libraries (Fig. 3). Of the clean distinct tags, only 2.2% (VDM) and 6.2% (VDMG-b) had more than 100 copies, 22.9% (VDM) and 29.5% (VDMG-b) had 5–50 copies, and 72.9% (VDM) and 60.2% (VDMG-b) had less than 5 copies (Fig. 3BD). All clean tags were aligned to the reference sequence of *V. dahliae* strain Vdls.17 isolated from an infected lettuce plant in California, USA (http://www.broadinstitute.org/annotation/genome/verticillium_dahliae). Of all the distinct tags, about 18.9% and 7.2% in the respective library of VDMG-b and VDM can be uniquely mapped to the reference sequence; about 57.9% and 52.6% of existing reference transcripts were matched by the tags in the VDMG-b and VDM library, respectively. Those tags mapped to the database generated 6,102 and 5,540 distinct tag-mapped transcripts for VDMG-b and VDM libraries, respectively. Most of the distinct tags (VDMG-b: 43.9%, VDM: 79.3%), however, cannot be aligned to the reference sequence due to incomplete sequence or a high genetic divergence between the two isolates (Table 3).

**Table 3:** Number of sequenced expression tags during the germination of *Verticillium dahliae* microsclerotia in the germinated (VDMG-b) and non-germinated (VDM) microsclerotia.

Summary		VDMG-b	VDM
Raw data	Total	3671123	3733211
	Distinct tag	252282	599603
Clean tags	Total number	3495080	3316376
	Distinct tag number	81034	186721
All tags mapped to genes	Total number	971836	770943
	Total % of clean tag	27.81%	23.25%
	Distinct tag number	15294	13397
	Distinct tag % of clean tag	18.87%	7.17%
Unambiguous tag mapped to genes	Total number	971074	769476
	Total % of clean tag	27.78%	23.20%
	Distinct tag number	15256	13361
	Distinct tag % of clean tag	18.83%	7.16%
All tag-mapped genes	Number	6130	5567
	Percentage of reference genes	58.19%	52.84%
Unique tag-mapped genes	Number	6102	5540
	Percentage of reference genes	57.92%	52.59%
Unidentified tags	Total number	964301	1506340
	Total % of clean tag	27.59%	45.42%
	Distinct tag number	35586	148084
	Distinct tag % of clean tag	43.91%	79.31%

Clean tags are tags after dirty tags (low quality tags) were excluded from the raw data. Unambiguous tags are the remaining clean tags after those tags that mapped to reference sequences from multiple genes were removed from the data.

**Figure 4:**
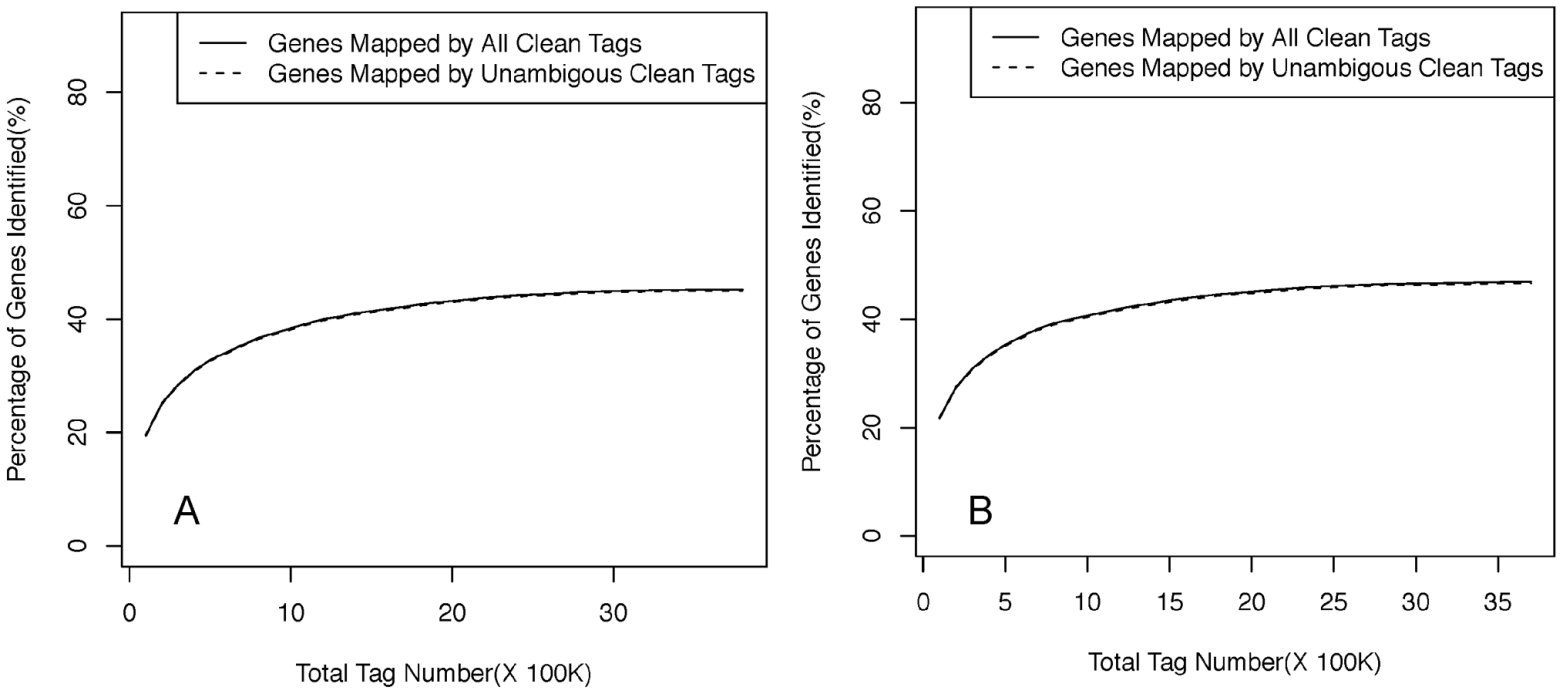
Percentage of total and unique clean tags that are mapped to the reference *Verticillium dahliae* genome VDLs 17 in the non-germinated (A) and germinated (B) microsclerotium libraries in relation to the total number of tags. New unique tag (y axis) of VDMG-b and VDM libraries decreased as the Illumina sequencing increased (x axis).

Sequencing saturation was analyzed for the two libraries to determine whether there is sufficient sequencing depth for transcriptome coverage. Number of genes mapped by unambiguous tags increased with the total number of tags in a similar way for both the libraries (Fig. 4). The number of detected genes was close to saturation when the number of mapped tags reached ca. 10^6^. About 9.7% (VDMG-b) and 2.9% (VDM) of the tags were matched by the distinct tags in the antisense direction, suggesting that these genes might be bidirectionally regulated. The frequency of the tags mapped on the antisense strand was generally much lower than on the sense strand.

### Differential gene expressions in the two libraries

**Figure 5:**
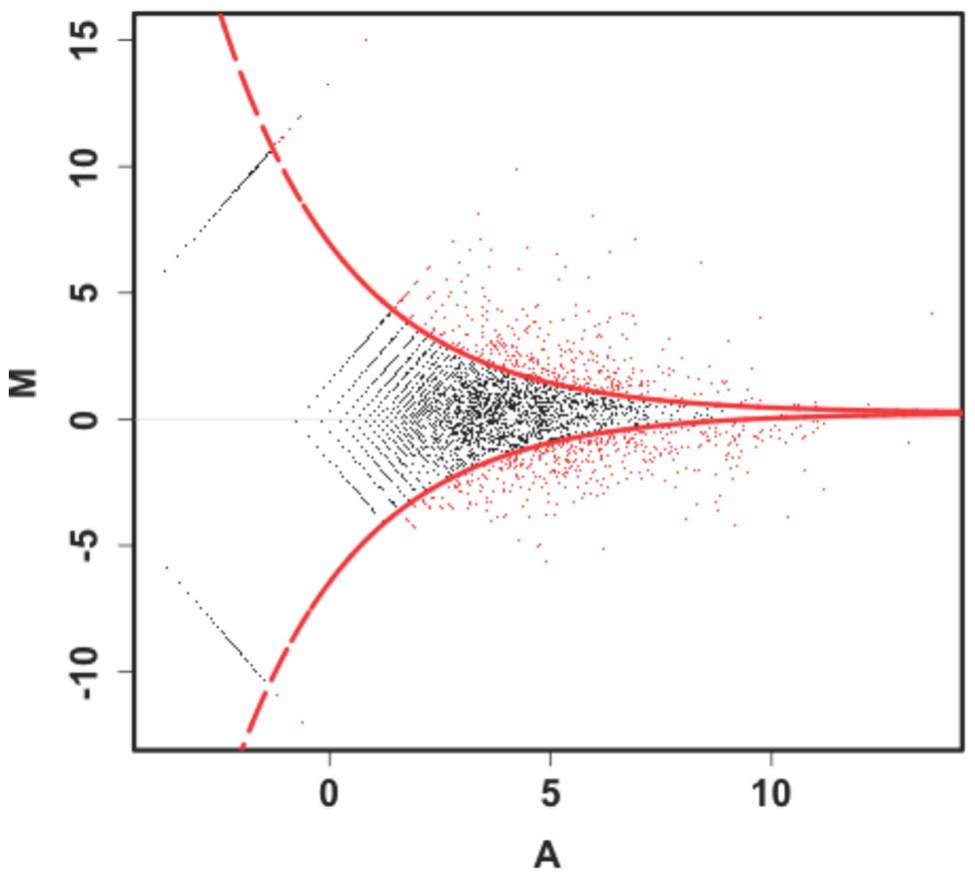
Comparision of the gene expression levels between the libraries of germinated (VDMG-b) *Verticillium dahliae* microsclerotia and non-germinated (VDM). Red dots represent differentially expressed genes in the VDMG-b library compared to VDM library using the Fisher's exact test at *P* = 0.001 and log2 Ratio ≥1.

**Figure 6:**
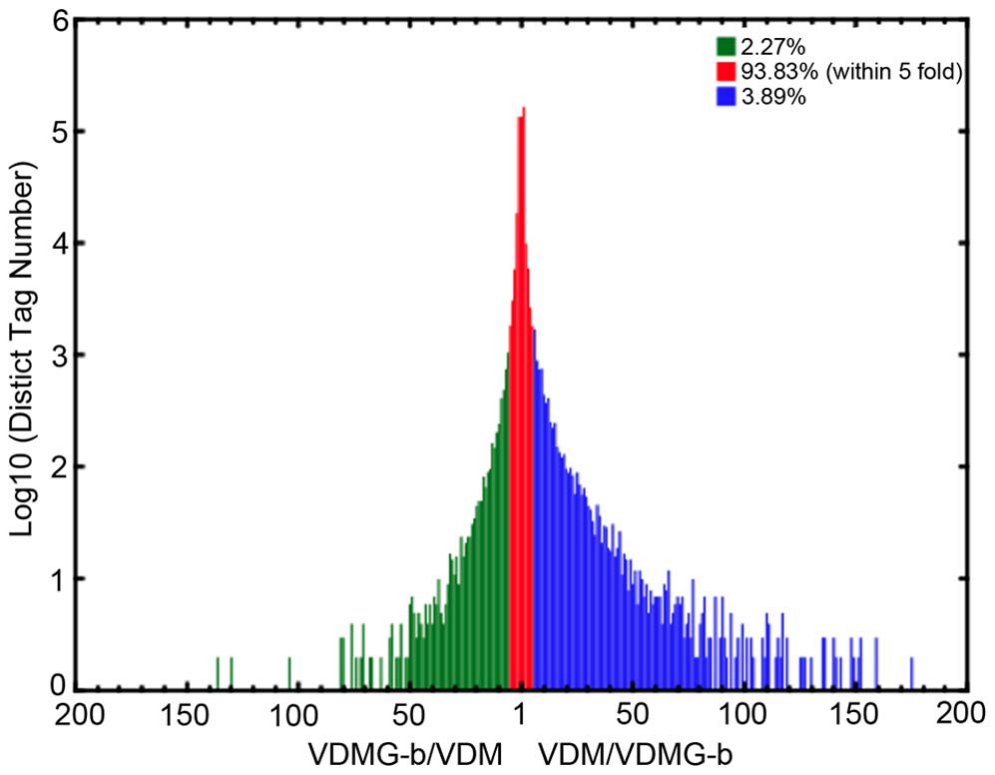
Frequency distribution of differentially expressed tags in the library of germinated *Verticillium dahliae* microsclerotium (VDMG-b) relative to the non-germinated microsclerotium library (VDM). The x axis represents the ratio of expression levels between the two libraries and the y axis represents the number of unique tags (log10).

A total of 1654 genes were differentialy expressed between the VDMG-b and VDM libraries, of which 925 and 729 were up- and down-regulated, respectively, in the VDMG-b library (Fig. S3). There were 781 and 606 genes that were up- and down-regulated with at least two-fold differences (FET *P*<0.001) in the expression level in VDMG-b library compared to VDM library (Fig. 5). The expression level was up-regulated and down-regulated by at least five-folds at about 3.9% and 2.3% of total unique tags, respectively, in the VDMG-b library (Fig. 6).

For the 40 most differentially expressed genes between the two libraries, 20 were up-regulated and 20 down-regulated in the VDMG-b library (Table 1). Several of the up-regulated genes in the VDMG-b library were associated with fungal infection, including genes encoding G-protein coupled receptor (GPCRs) and esterase; some were involved in the basic metabolism, including cyclopentanone 1,2-monooxygenase (CPMO), H(+)/hexose cotransporter 1, FAD binding domain-containing protein, fungal Zn(2)-Cys(6) binuclear cluster domain, thymus-specific serine protease, Zn_clus fungal specific transcription factor domain, glucan 1,3-beta-glucosidase, and alcohol dehydrogenase.

Among the 20 most down-regulated genes in the VDMG-b library (Table 1) included genes of putative pleiotropic ABC efflux transporter of multiple drugs, FAD binding domain-containing protein, mannose-6-phosphate isomerase, WSC domain-containing protein, vacuolar protein sorting-associated protein, glucan 1,3-beta-glucosidase, sulfate adenylyltransferase, pectin esterase, NADH-ubiquinone oxidoreductase 78 kDa subunit, cortical actin cytoskeleton protein asp1, mitochondrial phosphate carrier protein, SAP domain-containing protein, chitin deacetylase, and DNA damage-responsive transcriptional repressor RPH1. All of these genes are likely involved in the sugar metabolism, fat metabolism, sulfur metabolism, protein transport, and transcriptional regulation.

### Confirmation of tag-mapped gene expressions by qRT–PCR

**Figure 7:**
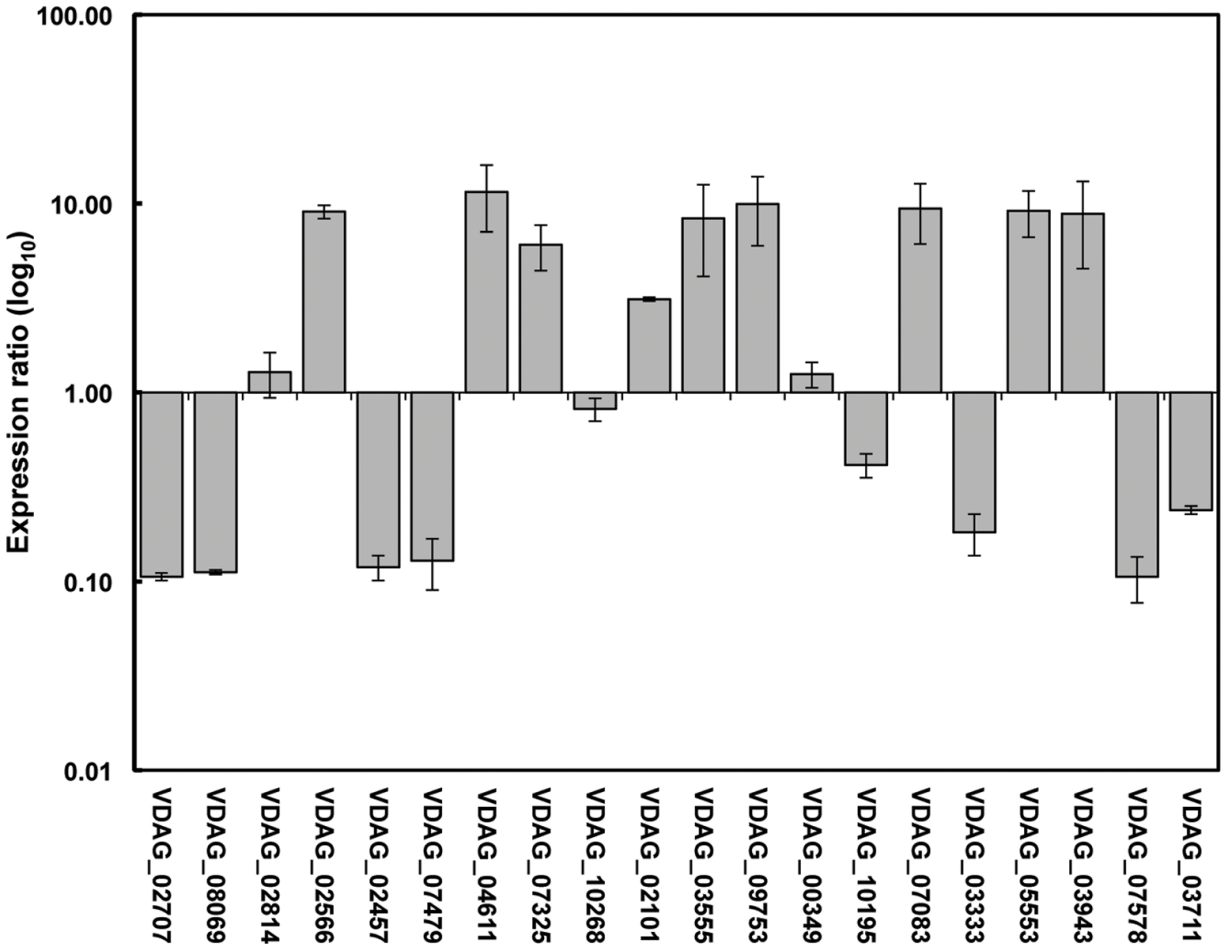
Relative expression level of 20 randomly selected genes from the 40 most differentially expressed genes that were up-regulated or down-regulated in the germinated *Verticillium dahliae* microsclerotia (VDMG-b) comapred with non-germinated *Verticillium dahliae* microsclerotia (VDM) by quantitative real-time reverse transpcription PCR. The expression ratio of each gene was calculated from cycle threshold (CT) values using 2^−ΔΔCT^ method [24]. Mean expression and error were determined using the REST (Relative Expression Software Tool) [25] with beta-tubulin gene as reference gene.

To confirm the tag-mapped differentially expressed genes between the two libraries, 20 genes were randomly selected from those 40 most differentially expressed genes in the VDMG-b library compared to the VDM library (ten up-regulated genes: VDAG_02814, VDAG-02566, VDAG-04611, VDAG-07325, VDAG-02101, VDAG-03555, VDAG-09753, VDAG-07083, VDAG-05553, and VDAG-3493; ten down-regulated genes: VDAG-02707, VDAG-08069, VDAG-02457, VDAG-07479, VDAG-10268, VDAG-00349, VDAG-10195, VDAG-03333, VDAG-07578, and VDAG-03711) for qRT–PCR assay. The expression level for 19 of the 20 genes was in agreement with the Tag-seq analysis (Fig. 7). The only exception is the SAP domain-containing protein encoding gene (VDAG_00349) where it was down-regulated in the Tag-seq analysis, but slightly up-regulated in the qRT-PCR assay. Relative gene expression levels of qRT-PCR were highly correlated with that of RNA-seq (R^2^ = 0.8534, *P*<0.01) (Fig. S4).

## Discussion

Microsclerotium germination of *V. dahliae* is the first developmental step in its pathogenesis. Germination must occur at the right time and in proximity to a suitable host for the fungus to infect plant roots. The mechanism(s) regulating microsclerotium germination is thus important for understanding to survival and pathogenesis microsclerotium in order to develop effective control methods.

In total, 6130 and 5567 tag-mapped genes were identified from the respective library of germinated and non-germinated *V. dahliae* microsclerotia on the basis of the reference sequence of *V. dahliae* strain VdLs.17. However, many tag-mapped genes cannot be identified from the published reference genome. This is not surprising given that the *V. dahliae* genome has not been completely sequenced yet. Many more genes may, therefore, be involved during germination of microsclerotia than those mapped onto the reference genome in the present study. These unmapped unique sequences may include novel genes that are needed to understand microsclerotium germination and pathogenesis.

Comparative analysis of gene expression levels suggested that at least 1654 genes were involved in microsclerotium germination. Notably, 104 genes were expressed exclusively in the germinating/germinated microsclerotia, and 34 genes were preferentially expressed in the non-germinated microsclerotia, suggesting that microsclerotia during the germination process undergo extensive transcriptional changes. Many genes that were highly expressed during the germination process were involved in cyclopentanone 1,2-monooxygenase (CPMO), G-protein coupled receptor (GPCR), lipase/esterase, amino sugar, nucleotide sugar, sulfur, and short chain alcohol metabolism. CPMO-associated gene showed the highest expression in the library of germinating or germinated *V. dahliae* microsclerotia. It has been most extensively studied in *Comamonas* sp. strain NCIMB 9872. Its function is to convert cyclopentanone to 5-valerolactone, which is dependent on NADPH and flavin adenine dinucleotide (FAD) [26]. CPMO is a very important enzyme in the body of organisms during metabolism, such as amino acids. CPMO from *Comamonas* (previously *Pseudomonas*) sp. strain NCIMB 9872 carries out the second step of a degradation pathway that allows the bacterium to use cyclopentanol as a sole carbon source for growth (United States Patent: 20050089846). GPCRs are a class of transmembrane receptors that sense molecules outside the cell and activate signal transduction pathways. GPCRs form the largest family of receptors in animals, with more than 600 members in the human genome [27], [28], and are important targets for animal drug discovery [29]. Therefore, identifying equivalent GPCRs in fungi may be similarly important for understanding the initial germination and subsequent pathogenesis phase in order to develop novel control methods. Krishnan et al. [30] identified 619 fungal specific GPCRs across 79 fungal genomes and revealed that Blastocladiomycota and Chytridiomycota phylum have Metazoan-like GPCRs rather than the GPCRs specific for fungi found only in Ascomycota and Basidiomycota [31], and provided the evidence on the presence of four of the five main GRAFS families in fungi. However, only a handful of GPCRs have been characterized in fungal genomes. For example, only three and four GPCRs receptors have been well studied in *Saccharomyces cerevisiae* and *Schizosaccharomyces pombe*, respectively [32]–[34]. In *Magnaporthe grisea*, the GPCR protein PTH11 contains the CFEM domain, which is required for pathogenesis [35]. Further studies are needed to assess whether GPCRs play a similar role in *V. dahliae*.

Genes coding esterase were also shown to express highly during the germination of microsclerotia. Esterase activity was found during germination of plant seeds [36] and fungal growth [37], but not found in crushed spores of *Aspergillus niger* [38]. In *A. niger*, esterase contributes to the degradation of plant cell walls by cleaving the diferulic cross-links between cell wall polymers [39]. The esterase can promote the hydrolysis of fats, a constitutent of plant cell walls, to provide energy. A high level of esterase activity might therefore help *V. dahliae* breakdown plant cell walls, and contribute to the infection of *V. dahliae* to host.

Understanding the initiation and regulation of microsclerotium germination may lead to development of novel disease control methods to disrupt this vital process necessary for pathogen infection and survival. The functions of these putative factors responsible for germination identified in the present study will be determined and verified by gene knockout or silencing mutants, which is now feasible since we have recently established a reliable transformation system for *V. dahliae*.

## Supporting Information

Figure S1 ***Verticillium dahliae*** **strain XJ2008 (AB551191) isolated from diseased cotton plant in Xinjiang province, China.** A, colony morphology on PDA; B, a single microsclerotium produced by strain XJ2008. C, branched conidiophores, which form whorls capped with flask-shaped and pointed phialides carrying terminal conidia.
(TIF)

Figure S2 **RNA-seq results of** ***Verticillium dahliae*** **microsclerotia.** A, histogram of the number of reads for genes in VDM library. B, histogram of the number of reads for genes in VDMG-b library. C, boxplot of read counts for each library. D, scatter plot comparing the number of reads for each gene between VDM and VDMG-b libraries.
(TIF)

Figure S3 **Number of differential expressed genes between the libraries of germinating/germinated (VDMG-b) and non-germinated (VDM)** ***Verticillium dahliae*** **.** Red and blue bars represent up- and down-regulated genes in VDMG-b compared to VDM, respectively.
(TIF)

Figure S4 **Comparison of expression levels determined by qRT-PCR and RNA-seq for the randomly selected 20 genes in VDMG-b library compared to VDM library.**
(TIF)

Table S1 **Experimental parameters for RNA-seq analyses of** ***Verticillium dahliae*** **microsclerotia.**
(DOC)
