# Supplementary figures and images for: Whole Genome Wide Expression Profiles on Germination of Verticillium dahliae Microsclerotia
Source: PLoS One. 2014 Jun 13;9(6):e100046. doi: 10.1371/journal.pone.0100046 (PMC4057337; doi:10.1371/journal.pone.0100046)

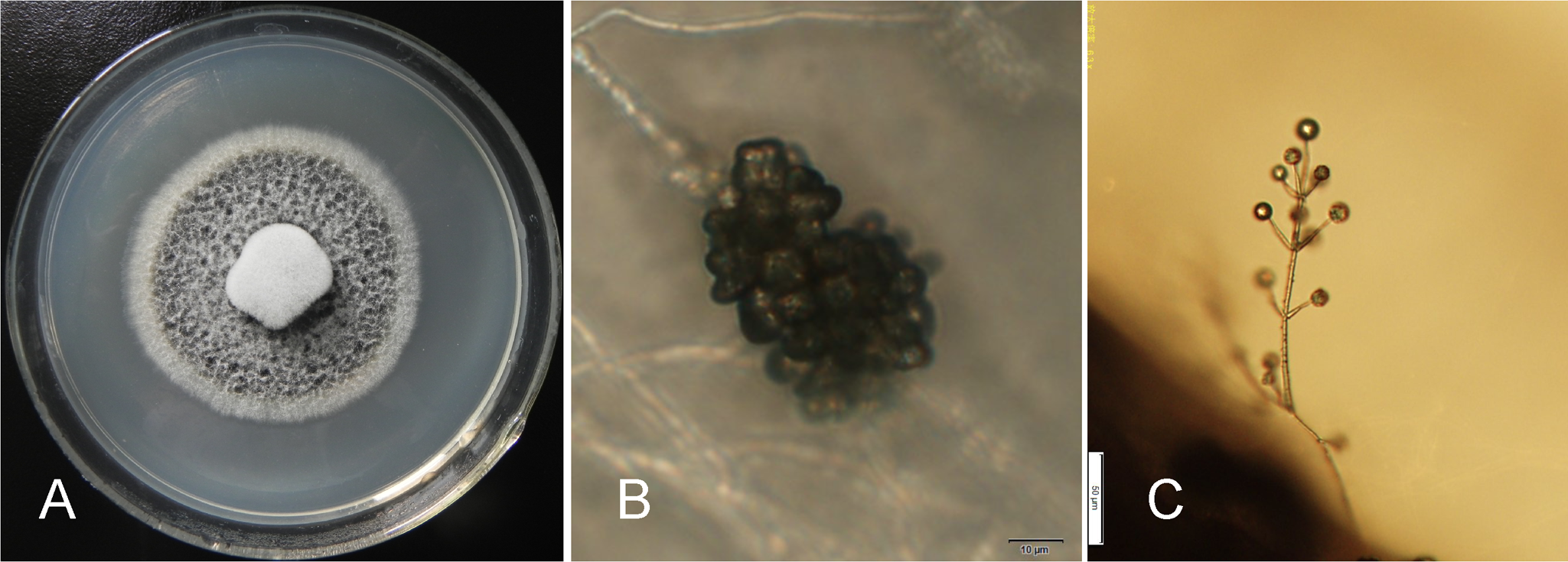

Supplement: Figure S1 — Verticillium dahliae strain XJ2008 (AB551191) isolated from diseased cotton plant in Xinjiang province, China. A, colony morphology on PDA; B, a single microsclerotium produced by strain XJ2008. C, branched conidiophores, which form whorls capped with flask-shaped and pointed phialides carrying terminal conidia. (TIF) [file pone.0100046.s001.tif]

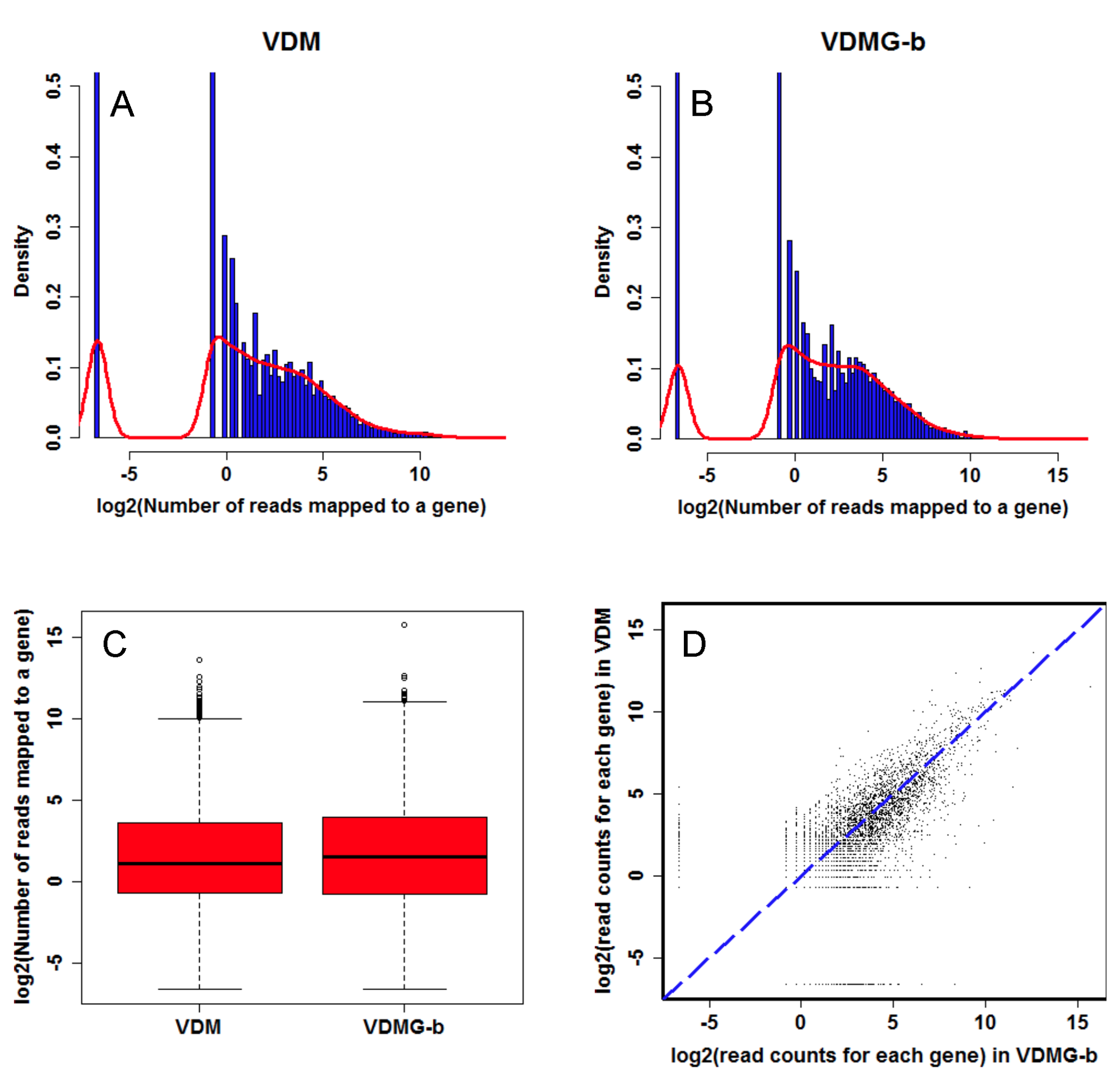

Supplement: Figure S2 — RNA-seq results of Verticillium dahliae microsclerotia. A, histogram of the number of reads for genes in VDM library. B, histogram of the number of reads for genes in VDMG-b library. C, boxplot of read counts for each library. D, scatter plot comparing the number of reads for each gene between VDM and VDMG-b libraries. (TIF) [file pone.0100046.s002.tif]

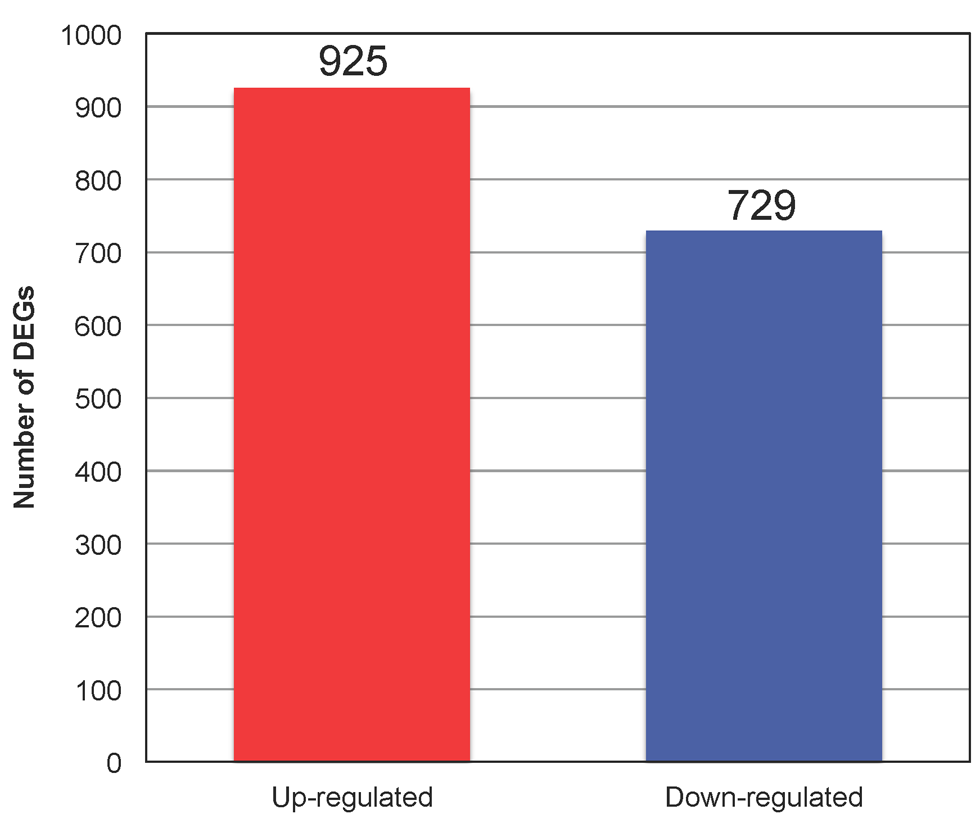

Supplement: Figure S3 — Number of differential expressed genes between the libraries of germinating/germinated (VDMG-b) and non-germinated (VDM) Verticillium dahliae . Red and blue bars represent up- and down-regulated genes in VDMG-b compared to VDM, respectively. (TIF) [file pone.0100046.s003.tif]

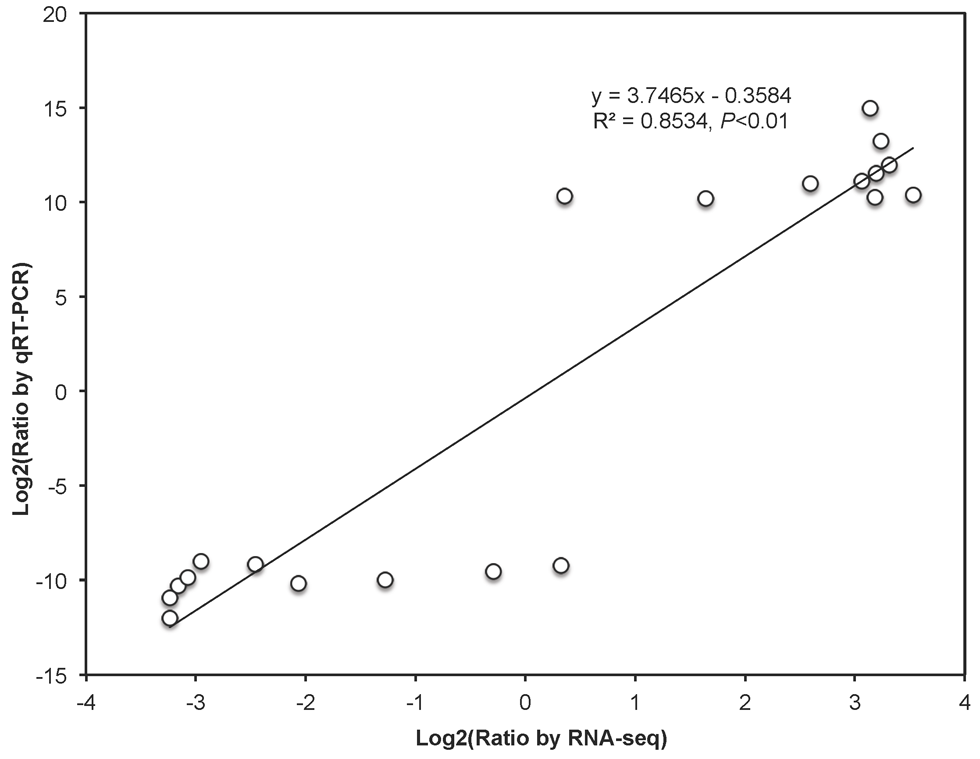

Supplement: Figure S4 — Comparison of expression levels determined by qRT-PCR and RNA-seq for the randomly selected 20 genes in VDMG-b library compared to VDM library. (TIF) [file pone.0100046.s004.tif]
