# Supplementary material for: Whole Genome Wide Expression Profiles on Germination of Verticillium dahliae Microsclerotia
Source: PLoS One. 2014 Jun 13;9(6):e100046. doi: 10.1371/journal.pone.0100046 (PMC4057337; doi:10.1371/journal.pone.0100046)
Supplement: Table S1 — Experimental parameters for RNA-seq analyses of Verticillium dahliae microsclerotia. (DOC) [file pone.0100046.s005.doc]

Table S1 Experimental parameters for RNA-seq analyses of *Verticillium dahliae* microsclerotia

| Sample name | RNA concentration  (ng/μl) | Volume  (μl) | Total  (μg) | RNA Integrity Number (RIN) | 28S:18S |
| --- | --- | --- | --- | --- | --- |
| VDM | 171 | 55 | 9.4 | 7.5 | 1.2 |
| VDMG-b | 227 | 40 | 9.1 | 6.1 | 1.0 |
